# Impacts of foreign direct investment on efficiency in Swedish manufacturing

**DOI:** 10.1186/s40064-016-2238-x

**Published:** 2016-05-12

**Authors:** Dick Svedin, Jesper Stage

**Affiliations:** Department of Business, Economics and Law, Mid Sweden University, 851 70 Sundsvall, Sweden; Department of Business Administration, Technology and Social Sciences, Luleå University of Technology, 971 87 Luleå, Sweden

**Keywords:** Foreign direct investment, Efficiency, Stochastic frontier analysis, Manufacturing, Sweden

## Abstract

A number of studies have found that foreign direct investment (FDI) can have positive impacts on productivity. However, while FDI has clearly positive impacts on technology transfers, its effects on resource use within firms is less clear and, in principle, efficiency losses might offset some of the productivity gains associated with improved technologies. In this paper, we study the impacts of FDI on efficiency in Swedish manufacturing. We find that foreign ownership has positive impacts on efficiency, supporting the earlier findings on productivity.

## Background

In this paper, we analyse whether the foreign ownership of manufacturing companies in Sweden has affected their technical efficiency, compared with their domestically owned (DO) counterparts. Efficiency measurement potentially captures a broader range of changes within firms than the more traditional productivity measurement does, but despite their obvious importance, the efficiency impacts of foreign direct investment (FDI) remain less well explored.

The fact that productivity increases when companies become foreign-owned (FO) is widely known (see e.g. JBIC 2002 for a review of literature in the field). There is, however, less analysis of the effects of foreign ownership on technical efficiency. In general we can say that, when a firm becomes more technically efficient, it automatically also becomes more productive. However, the reverse does not hold, i.e. increased productivity does not automatically cause higher technical efficiency. Productivity can improve either because available resources are used more efficiently, given the existing technology, because the technology itself improves, or (if measured as output per worker) because the amount of capital per worker increases. It is well known that foreign investors can bring in new technologies; however, given the various constraints that a foreign owner faces in comparison with a domestic owner, there is a risk that part of the productivity gain due to improved technology might be offset by reduced efficiency. If this happens, a country which seeks to attract FDI because of the anticipated benefits may in fact be foregoing some of these benefits without recognizing it, because the efficiency losses are masked by gains in other areas.

Until the early 1990s, FDI and foreign ownership were scarce in Sweden (Henrekson and Jakobsson 2005). This was partly due to a range of regulations. Before the 1990s, Sweden had a restrictive approach to FDI, with overlapping public and private rules and regulations as well as formal barriers to such investment. These measures included laws that allowed Swedish companies to restrict foreign ownership, laws that required foreign investors to apply for permission to acquire a Swedish company, a system of consent and strict practice (OECD 1993), and the regulation of foreign exchange flows. These measures were abolished around 1992,^1^ resulting in a significant inflow of FDI from then onward. Current Swedish policy is to encourage FDI, precisely because of the perceived benefits that foreign investors can bring; the Swedish foreign ministry has a specific division whose task it is to encourage foreign investors.

In 1980, just over 5 % of all workers in Sweden were employed by FO companies. By 2005, the proportion of workers employed in FO firms had jumped to almost 25 % of the country’s total employees. This corresponds to about 100,000 employees in 1980 and about 550,000 in 2005 (see Table 4 in Appendix).

The discussion in the rest of the paper is structured as follows: the second section provides a brief background on previous literature studying the impacts of FDI on productivity and efficiency. The third section presents the empirical method, namely the Stochastic Frontier Analysis approach, and the model specification used. The fourth section provides an overview of the data used in the paper, while the fifth section presents the results of the analysis. In the concluding section, the results are discussed.

## Previous literature

Foreign ownership affects companies in host countries in many different ways. These include impacts on the setting of wages, negotiating employment terms, spill-over effects, and productivity. In this review section, we will focus on studies investigating whether foreign ownership affects productivity and efficiency.

An extensive literature exists on productivity and foreign ownership. For example, a number of studies have shown that productivity in manufacturing companies has increased when such companies are taken over by foreign owners. In addition, these studies show that increases in productivity have been significantly higher for FO companies than for their DO counterparts.

The motives for a foreign investor to invest abroad are discussed by Girma et al. (2005), who argue that only the most productive firms find it profitable to meet the higher costs associated with FDI. In an earlier study, Girma et al. (2001) showed that, in the United Kingdom (UK), labour productivity was 10 % higher in FO firms in the first half of the 1990s, while the total factor productivity was 5 % higher in FO than DO firms. Harris and Robinson (2002), in their study on companies operating in the UK during the period 1974–1995, showed that foreign owners “cherry-picked” highly productive enterprises to invest in; their study also revealed that FO firms were 40 % more productive than their DO equivalents. Salis (2008) found similar results using Slovenian data for the 1994–1999 period. In a Norwegian study, Balsvik and Haller (2010), using data from between 1992 and 2004, established that FO companies selected “cherries” and managed to improve them further, while “lemons” were left to new DO buyers that seemed unable to do more than bring performance back to pre-acquisition levels. Results from an Italian study by Benfratello and Sembenelli (2006), who investigated manufacturing firms operating there between 1992 and 1999, revealed that the average FO firm was more likely to operate in high-tech industries, and was more productive than a DO firm. Ford et al. (2008), using aggregate data from 48 states in the United States (US) between 1978 and 1997, similarly found that FO firms outperformed DO firms in respect of productivity.

Studies based on Swedish data showed that DO firms also increased their productivity levels when they passed into foreign ownership. Hansson et al. (2007) showed that a positive correlation existed between foreign ownership and increased productivity. However, although the positive productivity effects of multinational ownership remained, they were weaker when one took the industrial sector and other controllable factors into account. This outcome of the study suggested there were structural, owner-specific reasons for the higher productivity. Another Swedish study, conducted by Modén (1998), also showed that Swedish companies increased their productivity when they passed into foreign hands. In the case of acquisitions, the investigation by Bandick and Karpaty (2011) revealed that Swedish companies exhibited 8 % higher total factor productivity, on average, after being acquired by foreign investors, in comparison with companies solely in Swedish possession.

Multinational companies can transfer foreign knowledge and foreign methods of production that DO firms do not have as easy access to. The evidence suggests that multinational firms employ more skilled workers (Görg and Greenaway 2004) and produce more advanced products (Kokko et al. 2001). Driffield and Love (2003) show that multinational companies are more research- and capital-intensive than DO companies are.

However, the notion of *productivity* should not be confused with that of *efficiency*. While *productivity* is the ratio of a firm’s output to its input, *efficiency* takes the form of the ratio of observed output to maximum potential output obtainable from given input, the ratio of minimum observed potential input required to produce given output, or some combination of these two.

Thus, for example, according to Moran et al. (2005), foreign owners can use host resources more efficiently and, by way of spill-overs, foreign ownership of a host country business contributes towards making it more efficient than before. This is not a given, however. There are several functions in a company’s business that are duplicated when the firm is owned by foreigners, including marketing and reporting to local authorities (who may be hostile to foreign owners in practice, regardless of what the official policy is) as well as building up relationships with local staff and local providers. Markusen (2002) and Bürker et al. (2013) demonstrate that these costs are important aspects of multinational companies’ decision on whether or not to produce abroad, and these added costs could potentially reduce the efficiency of firms that become FO. On the other hand, an important role that FDI can play is to effectively improve competition in the local markets and, at the company level, this could lead to improved efficiency as well. Thus, while productivity can be expected to improve as a result of FDI, the impact on efficiency is less obvious. Helpman et al. (2004) and Girma et al. (2005) found that only the most productive firms choose to set up operations in foreign countries, while less productive firms prefer to simply expand production in their home country and either export more or (for even less productive firms) sell more domestically.

That only the most productive firms see a gain to FDI rather than exporting shows that the transaction costs involved in setting up operations in another country are a real concern. Benfratello and Sembenelli (2006) found that technology transfers to foreign subsidiaries only take place when there are large technology differences between the foreign owner and the subsidiary, and not when the technology differences are smaller. This suggests—again—that there are important transactions costs involved, and that the gains from technology transfers have to be large in order to make it worthwhile to overcome the costs involved. Ford et al. (2008), comparing impacts of FDI on productivity in different US states, found that the level of human capital in the recipient state mattered for the productivity impact, again suggesting that conditions in the recipient area (other than those of the subsidiary firm receiving the investment) are crucial.

The impacts of FDI and foreign ownership on efficiency, rather than productivity, have therefore been studied in a growing (albeit still smaller than that for productivity) literature. Li (2008) studied firms that expanded abroad and found that they tended to become less efficient, at least in an initial phase of their expansion. Banalieva et al. (2012), also studying impacts on multinational enterprises as a whole, find similar effects of foreign expansion; they also find that the efficiency losses are smaller if the FDI is aimed at countries that are already integrated economically with the firm’s home country. Kinda (2012), comparing efficiency impacts of FDI in several developing and emerging economies, found that the investment climate in the recipient country had a marked effect not only for the efficiency impact in the FO firms but also for the efficiency in the local firms selling to them. This suggests that whether FDI and FO firms will see improved efficiency or not will depend on the recipient country and may also depend on the recipient sector. Saranga and Phani (2009), studying efficiency in the Indian pharmaceutical industry, found that the FO firms tended to see efficiency improve, and Suyanto and Salim (2013) found similar results for Indonesian pharmaceuticals. On the other hand, when studying two different Indonesian manufacturing sectors (Suyanto and Salim 2010), they found that FDI led to increased efficiency in one sector but reduced efficiency in the other. Khalifah (2013), studying Malaysia’s automotive industry, found that FO firms were more efficient overall, but that this was not the case in all the component subsectors of the industry.

Whether FDI leads to improved efficiency, as opposed to “merely” increased productivity, is not merely an academic issue. Görg and Greenaway (2004) note that many countries, as well as regional and local jurisdictions, provide direct and indirect subsidies to foreign investors in the hope that this will attract productive companies to their jurisdictions. FO companies are indeed more productive than their DO counterparts, as the literature reviewed above indicates. However, if transaction costs linked to establishing foreign affiliates are important, in the sector or in the country as a whole, part of the productivity gains may be lost. If FO firms see reduced efficiency, the recipient countries forego some of the economic gains from FDI that they are trying to achieve; and if they observe only the productivity gains, they may not realise that those gains could have been even higher. It is therefore worthwhile to investigate whether the increased productivity observed for FO firms in Sweden is associated with reduced or increased efficiency, in order to ascertain whether the climate for foreign investors lets the country make full use of its potential gains from FDI. The aim of this paper, therefore, is to study whether foreign participation affects technical efficiency in Swedish manufacturing, and whether the effects vary by sector.

## Stochastic production frontier analysis

The model in the present paper is based on that devised by Battese and Coelli (1995) and can be described as follows. The stochastic production frontier function for panel data is assumed to be

1 $$y_{it} = f(x_{it} ;\beta )\exp \left\{ {\nu_{it} - u_{it} } \right\}$$

where *y*_*it*_ denotes the production at time *t*$(t = 1,2, \ldots ,T)$ of the *i*th firm $(i = 1,2, \ldots ,N)$, *x*_*it*_ is a (1 × *h*) vector of values of inputs of production and other explanatory variables associated with the *i*th firm at the *t*th observation, *β* is a (*h* × 1) vector of values of parameters to be estimated, *ν*_*it*_ is a random error and *u*_*it*_ is the technical inefficiency of the firm. The *ν*_*it*_s are assumed to be iid $N(0,\sigma_{\nu }^{2} )$ random errors, which are assumed to be independently distributed of the *u*_*it*_s. Thus, a firm with no technical inefficiency (*u*_*it*_ = 0) will have an output given by *f*(*x*_*it*_; *β*) times a random term exp(*ν*_*it*_) with expectation value one.

The *u*_*it*_s are non-negative random variables, associated with technical inefficiency of production, which are assumed to be independently distributed, such that *u*_*it*_ is obtained by truncation at zero of a normal distribution with mean $z_{it} \delta$ and variance *σ*^2^. The vector of explanatory variables, *z*_*it*_, has the dimension (1 × *m*) where *δ* is a (*m* × 1) vector of unknown coefficients. The technical inefficiency term *u*_*it*_ in the stochastic frontier in model Eq. (1) can be written as

2 $$u_{it} = z_{it} \delta + w_{it}$$

where *w*_*it*_ is a random variable which is defined by a truncation of the normal distribution with zero mean and variance *σ*^2^, so that the truncation point is $- z_{it} \delta$, i.e. $w_{it} \ge - z_{it} \delta$. This assumption is consistent with *u*_*it*_ being a non-negative truncation for $N( - z_{it} \delta ,\sigma^{2} )$. The assumption that *u*_*it*_ and *ν*_*it*_ are independently distributed for all *t* = 1, 2, …, *T* and *i* = 1, 2, …, *N* is a simplifying, but obviously also relatively restrictive, condition. Battese and Tessema (1993) suggest applying the method of maximum likelihood for simultaneous estimation of the parameters in the stochastic frontier model and in the inefficiency model.

The technical efficiency (*TE*) of production for the *i*th firm at the *t*-th observation is therefore defined by

3 $$TE_{it} = \exp \left\{ { - u_{it} } \right\} = \exp \left\{ { - z_{it} \delta - w_{it} } \right\}$$

The prediction of the technical inefficiency is based on its conditional expectation, given the model assumptions (Battese and Coelli 1992).

The stochastic frontier of the production function is estimated as a standard translog production function with production determined by capital input *k* and labour input *l*, and with coefficients potentially changing over time:

4 $$\begin{aligned} \log y_{it} & = \beta_{0} + \beta_{k} \log k_{it} + \beta_{l} \log l_{it} + \beta_{t} t \\& \quad + \frac{1}{2}\beta_{kk} (\log k_{it} )^{2} + \beta_{kl} \log k_{it} \log l_{it} + \beta_{kt} \log k_{it} t \\& \quad + \frac{1}{2}\beta_{ll} (\log l_{it} )^{2} + \beta_{lt} \log l_{it} t + \frac{1}{2}\beta_{tt} t^{2} \\& \quad + \nu_{it} - u_{it} \\ \end{aligned}$$

With this setup, we see that it is possible for a firm to increase its productivity over time but simultaneously see its inefficiency increase, the potential outcome that concerns us for the FO firms. The net outcome might then still be an increase in overall production, but a smaller increase than would have occurred if inefficiency had remained constant or decreased. Including foreign ownership as one of the determinants of *u* lets us see whether foreign ownership affects efficiency positively or negatively.

Since each industry can be assumed to have its own technology, the model is estimated separately for each industrial sector, defined at the three-digit standard industrial classification (SIC) level. However, a pooled model for the entire manufacturing sector is also estimated.

Differentiating log*y*_*it*_ with respect to $\log k_{it}$ and $\log l_{it}$, respectively, gives us the production elasticities with regard to capital and labour. Taking the sum of both elasticities lets us measure returns to scale, *RTS*. *RTS* is expected to be approximately 1 for most sectors; the two elasticities are expected to be greater than zero but less than one for all sectors, but may vary considerably between different sectors.

5 $$e_{it}^{k} = \beta_{k} + \beta_{kk} \log k_{it} + \beta_{kl} \log l_{it} + \beta_{kt} t$$

6 $$e_{it}^{l} = \beta_{l} + \beta_{ll} \log l_{it} + \beta_{kl} \log k_{it} + \beta_{lt} t$$

7 $$RTS = e_{it}^{k} + e_{it}^{l}$$

Differentiating with respect to *t* gives us the rate of technical change, *TC*, which is expected to be on the order of a few per cent per year.

8 $$TC = \beta_{t} + \beta_{kt} \log k_{it} + \beta_{lt} \log l_{it} + \beta_{tt} t > 0$$

The technical inefficiency effects are assumed to be defined by

9 $$u_{it} = \delta_{0} + \delta_{FO} FO_{it} + \delta_{k/l} (k/l) + \delta_{k \cdot l} (k \cdot l) + \delta_{t} t + \delta_{D92} D92_{it} + \varepsilon_{it}$$

where ownership is a vital variable to incorporate in the efficiency function in the present paper, since different owners are assumed to behave differently when it comes to managing. We only consider different management with respect to the relevant owner’s domicile, i.e. whether the firm is DO or FO. This was done using a dummy variable for FO firms (where FO = 0 if it is a DO firm, and where FO = 1 if it is an FO firm). A positive sign for $\delta_{FO}$ would imply that FO firms are more inefficient than DO firms, while a negative sign would imply the opposite. The *k*/*l* term, which measures capital intensity, is included in order to explain whether or not high capital intensity affects efficiency, whereas *k* × *l*, which measures the cross-elasticity of capital and labour, is included so that we can see whether economies of scale affect efficiency. We also include a general time trend *t*, as well as a 1992 dummy which is used for controlling whether management practices changed after the turbulence of 1992. *ɛ*_*it*_, finally, is a random variable. There are no a priori expectations from theory for any of the coefficients in the inefficiency equation.

## Data

The data we use is a panel data set for manufacturing firms in Sweden compiled by one of the authors (Brännlund et al. 2016). The panel covers the years 1980 to 2005, and consists of all manufacturing firms with at least 50 employees (as most FO firms have more employees than this, this helps ensure greater comparability between DO and FO firms; data errors are also more frequent among the smaller firms). Table 3 in the Appendix offers a classification of the industries. Since the classification of industries changed during the period studied, only firms that belong to the same industry in both classification systems (SNI69 and SNI92) are included. To be classified as an FO company, foreigners had to have more than 50 % of the votes in the company. Most of the variables were collected from each firm’s annual report, obtained from the Swedish Registrar of Companies. The information on the main owner’s origin was collected from each firm’s record of stock ownership at the time of the shareholders’ annual general meeting.

Several criteria were used to select firms for the study from the full data set. Firstly, in order for a firm to be included in the data set, it had to have at least 50 employees. Secondly, production had to be relatively homogeneous (which reduced the sample sharply). Thirdly, the firm had to have started its activity before 1992 (as noted, this year was important for controlling whether management practices had changed in the firm after the turbulence of 1992). Fourthly, the firm had to have at least 5 years of continuous activity (which makes it possible to study its operations for a longer period). These criteria gave us a high share of Swedish-owned firms (nearly 50 %). The second-largest owner of firms in the data set was Finland: 8.5 % of all firms had Finnish owners during the period in question. In total, the data set consists of 242 firms that meet all of the above criteria for inclusion in the analysis.

Output is measured in real 1980 SEK. Labour input is measured as the number of employed individuals in the firm in the year in question, while the capital stock is measured as the real value of physical capital (machinery, equipment and buildings) in the firm. Average productivity during 1980–2005 among the firms that are included in the data set (Table 5 in Appendix) was 767,670 SEK per employee and year in constant 1980 prices. The average value of real capital was 145,383 SEK in 1980 prices, while the average number of employees was 314.

## Results

Two-sample *t* tests (see Tables 7, 8, 9 in the Appendix for details) show that, on average, FO companies had more employees, larger capital stocks and higher productivity than Swedish-owned companies did. All three tests were significant at the 1 % level. Thus, the results confirm the finding that FO companies tend to have higher productivity per employee. However, the capital stock per employee is also greater; this explains at least some of the productivity difference, and thus investigating whether the FO companies use their resources more efficiently remains of interest.

The stochastic production function was estimated as a translog function using a maximum likelihood (*ML*) estimator. Table 1 presents the estimated parameters.

**Table 1:** Maximum likelihood estimates for parameters of the inefficiency function for six manufacturing industries in Sweden and a pooled estimate, 1980–2005

Variable	Pooled, all sectors	Forest	Beverage	Chemical	Concrete	Metal	Electro
*Stochastic production frontier*							
*β*_*k*_	0.5632* (−0.0286)	0.1673 (0.1312)	0.1250 (0.1650)	0.3626* (0.0357)	0.4134* (0.0455)	1.0031* (0.0675)	0.7984* (0.0599)
*β*_*l*_	0.3686* (0.0337)	0.6423* (0.1442)	0.3194** (0.1528)	0.5031* (0.0659)	0.5789* (0.0921)	0.1126 (0.0704)	−0.0349 (0.0940)
*β*_*t*_	0.0185 (0.0102)	−0.0367** (0.0155)	0.0280 (0.0218)	0.0234* (0.0068)	0.0576* (0.0145)	−0.0178 (0.0106)	−0.0161 (0.0101)
*β*_*kk*_	0.1869* (0.0178)	0.0206 (0.0765)	−0.0252 (0.1088)	0.1680* (0.0198)	0.2665* (0.0694)	0.3849* (0.0345)	0.1605* (0.0324)
*β*_*kl*_	−0.1686* (0.0196)	−0.0876 (0.0810)	0.0104 (0.1310)	−0.2489* (0.0437)	−0.2252** (0.0934)	−0.2712* (0.0360)	0.0233 (0.0429)
*β*_*kt*_	−0.0076* (0.0012)	−0.0078* (0.0029)	−0.0210** (0.0105)	0.0001 (0.0021)	0.0116** (0.0047)	−0.0187* (0.0028)	−0.0175* (0.0033)
*β*_*ll*_	0.0880* (0.0318)	0.0233 (0.0931)	−0.4320** (0.2034)	0.4614* (0.0910)	0.4670* (0.1334)	0.0192 (0.0603)	−0.3437* (0.1112)
*β*_*lt*_	0.0096* (0.0017)	0.0101 (0.0038)	0.0192 (0.0104)	−0.0080 (0.0043)	−0.0013 (0.0061)	0.0183* (0.0036)	0.0214* (0.0057)
*β*_*tt*_	0.0010* (0.0003)	0.0025* (0.0005)	0.0040 (0.0017)	0.0002 (0.0005)	0.0074* (0.0019)	0.0016* (0.0006)	0.0035* (0.0007)
Constant	−0.0311 (0.1065)	1.4077* (0.2698)	0.5334* (0.1691)	−0.2378* (0.0567)	−1.0186* (0.0678)	0.1270 (0.1082)	0.4172* (0.0830)
*Technical inefficiency model*							
*δ*_*FO *_	−0.2117* (0.0217)	−0.3954* (0.0296)	−0.4565* (0.0956)	0.9094 (0.5863)	0.0140 (0.0580)	−0.0230 (0.1407)	−1.1771* (0.3475)
*δ*_*k*/*l*_	0.0814* (0.0116)	−0.0609 (0.1169)	−0.4034** (0.1813)	3.1548** (1.6092)	0.1460* (0.0389)	0.4836* (0.0757)	0.3005 (0.1676)
*δ*_*k*·*l*_	−6.08 × 10^−08^ (3.21 × 10^−08^)	−0.0185* (0.0033)	−0.1065* (0.0268)	−1.5313 (0.7998)	0.0486** (0.0221)	1.29 × 10^−07^ (1.37 × 10^−07^)	−0.3366* (0.1223)
*δ*_*t*_	0.0186 (0.0108)	−0.0105 (0.0125)	0.0976* (0.0277)	0.0602 (0.0418)	0.1789* (0.0272)	−0.0371** (0.0160)	−0.0111 (0.0128)
*δ*_92_	0.0955* (0.0340)	0.0432 (0.0609)	−0.0758 (0.1674)	0.1005 (0.5899)	0.2264** (0.0943)	0.4184** (0.1883)	0.4854 (0.2839)
Constant	0.1396 (0.1133)	1.7364* (0.3262)	0.9621* (0.2639)	−6.6931 (4.1312)	−1.9864* (0.3176)	−0.5043 (0.2720)	0.6073* (0.2297)
*σ*_*u*_^2^	4.5104* (0.9421)	2.8196* (0.1143)	1.1059* (0.1778)	0.4782 (0.6265)	2.8830* (0.2429)	2.2581* (0.3601)	1.0327* (0.2834)
*σ*_*v*_^2^	1.6786* (0.0559)	5.4708* (1.2117)	2.2791* (0.4303)	2.3666* (0.0672)	2.9171* (0.1453)	1.4705* (0.0471)	3.8270* (0.3267)
Log-likelihood	−2592.36	−12.69	−403.1	−380.88	−71.39	−1155.52	−87.13
Number of observations	4277	473	434	1023	377	1613	357
Number of cross-sections	240	25	21	59	19	90	20
Number of time periods	26	26	26	26	26	26	26
Average number of time periods	18	19	21	17	19	18	17

Standard errors in brackets

Significance: * = 0.1 % level, ** = 1 % level, and *** = 5 % level

The stochastic production frontier model estimates in Table 1 indicate that for each industry, as well as for the pooled model, the parameters are in line with the theoretical expectations outlined in the previous section. All estimated elasticities for capital and labour (see Table 2) have reasonable values except for the capital elasticity for the *Electro* industry, which is not statistically significant. Returns to scale are below 1 for the pooled model as well as for most of the sector-level models, and for the one sector where it is greater than one it is not statistically significantly so. The technical change coefficients are all positive and of the expected magnitude, although not statistically significant for all sectors.

**Table 2:** Elasticities

	Pooled	Forest	Beverage	Chemical	Concrete	Metal	Electro
*ɛ*_*k*_	0.3806 (0.1567)	0.1089 (0.0366)	0.2204 (0.1204)	0.1113 (0.0781)	0.2607 (0.677)	0.1198 (0.0980)	−0.0117 (0.1666)
*ɛ*_*l*_	0.6004 (0.1683)	0.8014 (0.0832)	0.7080 (0.1138)	0.7619 (0.0645)	0.6473 (0.0983)	0.8848 (0.0537)	0.9042 (0.2121)
Returns to scale	0.9106 (0.0572)	0.9103 (0.1018)	0.9284 (0.0793)	0.8733 (0.0671)	0.9080 (0.0691)	1.0046 (0.0950)	0.8925 (0.0674)
Rate of technical change	0.0266 (0.0061)	0.0150 (0.0138)	0.0181 (0.0185)	0.0278 (0.0051)	0.0303 (0.0179)	0.0254 (0.0075)	0.0544 (0.0275)
No. of observations	4277	473	434	1021	775	1603	337

Standard errors in brackets

In the inefficiency model, we see that an increase in capital intensity sees a concomitant increase in inefficiency in all except the *Beverage* and *Forest* industries, and that it also increases inefficiency in the pooled model. On the other hand, when the scale increases, inefficiency declines in the *Forest*, *Beverage* and *Electro* sectors—for all of them significantly so. The time trends for inefficiency are insignificant except for the *Beverage*, *Concrete* and *Metal* industries, which become more inefficient over time. Moreover, the dummy variable for the year 1992 indicates significantly higher inefficiency from 1992 onwards for the *Concrete* and *Metal* sectors.

Looking at foreign ownership, the focus of our study, the significantly negative results in the pooled model and for the *Forest*, *Beverage* and *Electro* sectors indicate that, in those industries, foreign ownership improves efficiency. The tests of impacts of foreign ownership for the *Chemical*, *Concrete* and *Metal* industries are all insignificant. For the pooled model, the dummy variable for foreign ownership is negative, which indicates that, for the sample as a whole, firms with foreign owners become less inefficient. There is no sector where there is a statistically significant increase in inefficiency linked to foreign ownership.

## Conclusions

The main purpose of this paper was to investigate whether foreign ownership affects Swedish manufacturing firms’ technological efficiency. Our results indicate that inefficiency in Swedish companies is affected by whether their owners are non-Swedish or Swedish: FO firms, taken as a whole, are less inefficient, and this remains true when studied at the sectoral level. For some sectors, there is a statistically significant decrease in inefficiency linked to foreign ownership, while for the others, there is no statistically significant effect at all. Thus, most of the FO firms seem to be either as inefficient as their DO counterparts, or less.

Previous studies on the foreign ownership of Swedish manufacturing firms have concluded that such companies become more productive when they are acquired by foreign owners; similar results have been found for other countries. However, since foreign ownership tends to bring with it better access to new technologies, productivity increases linked to better technologies might mask reduced resource efficiency linked to a more limited understanding of the local context. Thus, studying inefficiency gives us more informative results than productivity studies alone would. By examining the inefficiency in a firm, we find evidence that FO companies are systematically more efficient than DO firms in some, but not all, sectors. Thus, the exact impact of foreign ownership on productivity and efficiency is potentially less clear-cut than earlier studies have indicated, and the exact pathway through which foreign ownership affects resource use within firms deserves further study.

Nonetheless, one implication of these findings is that the shift in Swedish policy in the early 1990s, from discouraging foreign investors to encouraging them, appears to be working as intended. For those sectors where an owner-specific effect on efficiency is at all discernible, the effect of foreign ownership is to reduce inefficiency. As noted in the literature review, foreign investors in other countries have frequently found that transactions costs associated with locating part of their production away from their home country reduce the efficiency of their operations, reducing the productivity gains from foreign ownership. We find no such effect for any of the Swedish sectors studied in this paper.

## Appendix

See Tables 3, 4, 5, 6, 7, 8 and 9.

**Table 3:** Standard industrial classification (SIC) codes

Code	SIC code	Industries
D101	154,155 and 158	Manufacture of Vegetable and animal oils and fats Dairy products, and Other food products
D102	211	Manufacture of pulp, paper and paperboard
D134	241, 243,245 and 246	Manufacture of Basic chemicals Paints, varnishes and similar coatings, printing ink and mastics Soap and detergents, cleaning and polishing preparations, perfume and toilet preparations, and Other chemical products
D105	261	Manufacture of glass and glass products
D106	265, 266 and 268	Manufacture of Cement, lime and plaster Articles of concrete, plaster and cement, and Other non-metallic mineral products
D178	272, 273, 274, 282 and 283	Manufacture of Tubes Other first processing of iron and steel Basic precious and non-ferrous metals Tanks, reservoirs and containers of metals, central heating radiators and boilers, and Steam generators, except central heating hot water boilers
D109	286	Manufacture of cutlery, tools and general hard ware
D110	291 and 295	Manufacture of Machinery for the production and use of mechanical power, except aircraft, vehicle and cycle engines, and Other special purpose machinery
D111	313 and 314	Manufacture of Insulated wire and cable, and Accumulators, primary cells and primary batteries

**Table 4:** Number of employees in foreign-owned firms and the share of employed in Swedish manufacturing industry between 1980 and 2010

Year	Number of employees	Share of employment (%)	Growth of employment in foreign-owned firms (%)
1980	113,998	5.4	
1981	125,928	5.9	10.5
1982	127,305	6.2	1.1
1983	131,832	6.5	3.6
1984	124,639	6.2	−5.5
1985	139,737	6.9	12.1
1986	148,378	7.0	6.2
1987	154,217	7.2	3.9
1988	192,629	9.0	24.9
1989	201,970	8.9	4.8
1990	206,886	8.7	2.4
1991	228,713	9.7	10.6
1992	222,062	9.9	−2.9
1993	210,252	10.0	−5.3
1994	214,014	10.5	1.8
1995	246,018	11.7	15.0
1996	278,016	12.9	13.0
1997	301,069	13.9	8.3
1998	333,395	15.0	10.7
1999	397,665	16.9	19.3
2000	446,893	19.0	12.4
2001	520,081	21.4	16.4
2002	530,758	21.7	2.1
2003	564,180	23.2	6.3
2004	544,579	23.0	−3.5
2005	557,496	22.9	2.4

**Table 5:** Descriptive statistics: employees, real wage, real capital stock and producer price index

Year	No. of employees	Real wage	Real capital	Producer price index	Productivity
1980					
Average	380.670	102.015	120.419	1.000	527.677
SD	458.088	16.694	198.044	0.000	467.661
N	140	140	140	140	140
1981					
Average	362.772	96.779	108.853	1.100	528.211
SD	429.298	16.144	153.160	0.059	555.370
N	142	142	142	142	142
1982					
Average	348.964	107.327	100.325	1.248	531.286
SD	409.256	18.125	120.072	0.091	586.822
N	145	145	145	145	145
1983					
Average	321.640	123.549	94.516	1.383	554.124
SD	375.437	28.233	97.095	0.093	646.312
N	161	161	161	161	161
1984					
Average	343.937	134.405	96.264	1.459	570.520
SD	394.163	33.501	114.198	0.176	637.916
N	163	163	163	163	163
1985					
Average	338.498	150.485	101.642	1.634	607.034
SD	392.101	36.783	107.794	0.148	616.439
N	175	175	175	175	175
1986					
Average	339.335	168.176	107.425	1.567	608.682
SD	395.867	48.242	111.470	0.147	566.676
N	182	182	182	182	182
1987					
Average	330.091	177.979	114.492	1.591	608.608
SD	380.371	46.432	115.814	0.200	490.681
N	183	183	183	183	183
1988					
Average	327.784	186.348	115.364	1.683	632.459
SD	391.832	34.749	121.198	0.257	596.536
N	188	188	188	188	188
1989					
Average	327.684	212.848	115.339	1.823	643.367
SD	404.388	42.121	107.256	0.277	632.380
N	196	196	196	196	196
1990					
Average	308.922	222.191	126.175	1.890	666.083
SD	377.647	42.114	117.663	0.227	566.517
N	206	206	206	206	206
1991					
Average	304.545	227.469	130.401	1.919	639.876
SD	370.322	53.231	115.357	0.239	591.731
N	199	199	199	199	199
1992					
Average	281.522	269.071	145.968	1.900	657.930
SD	348.292	50.193	132.164	0.242	577.893
N	203	203	203	203	203
1993					
Average	272.443	262.533	149.437	1.978	727.018
SD	347.710	73.071	129.173	0.237	715.529
N	193	193	193	193	193
1994					
Average	273.926	291.073	146.483	2.087	838.569
SD	343.040	70.973	118.811	0.232	842.387
N	190	190	190	190	190
1995					
Average	293.260	312.103	153.163	2.307	858.497
SD	372.044	63.009	126.385	0.287	835.354
N	179	179	179	179	179
1996					
Average	305.206	352.906	201.958	2.291	893.090
SD	410.042	247.275	573.254	0.309	1173.129
N	180	180	180	180	180
1997					
Average	300.294	358.009	216.978	2.304	1031.706
SD	395.837	61.249	598.871	0.296	1402.772
N	180	180	180	180	180
1998					
Average	308.691	372.644	174.755	2.290	890.661
SD	395.880	69.712	149.506	0.297	837.151
N	175	175	175	175	175
1999					
Average	309.928	386.083	182.305	2.260	899.479
SD	405.005	75.859	153.051	0.290	736.334
N	167	167	167	167	167
2000					
Average	306.335	392.655	184.352	2.341	957.271
SD	405.311	78.483	163.636	0.282	898.903
N	164	164	164	164	164
2001					
Average	303.425	428.677	182.998	2.452	970.732
SD	400.085	261.198	176.288	0.315	891.795
N	153	153	153	153	153
2002					
Average	289.283	427.755	197.718	2.505	1092.468
SD	392.218	80.294	183.278	0.312	1293.348
N	145	145	145	145	145
2003					
Average	304.121	450.876	180.178	2.449	1012.040
SD	433.587	98.539	165.626	0.332	860.525
N	141	141	141	141	141
2004					
Average	297.526	466.846	180.526	2.498	1069.349
SD	424.616	81.516	169.597	0.320	1016.827
N	135	135	135	135	135
2005					
Average	299.907	496.293	175.090	2.643	1178.552
SD	429.309	112.203	170.629	0.406	1063.831
N	129	129	129	129	129
Average					
Average	313.577	271.733	145.383	1.942	767.670
SD	393.360	147.857	215.975	0.500	821.586
N	4414	4414	4414	4414	4414

**Table 6:** Descriptive statistics: output, capital and labour by SIC code

SIC	Output	Capital	Labour
150			
Average	356,059	164,550	436
SD	449,901	241,820	465
N	435	435	435
212			
Average	278,006	131,457	443
SD	644,634	234,588	692
N	478	478	478
241			
Average	453,981	286,064	379
SD	504,375	517,519	379
N	277	277	277
243			
Average	170,275	46,878	209
SD	142,094	52,897	193
N	326	326	326
245			
Average	107,802	20,575	153
SD	86,663	24,158	118
N	151	151	151
246			
Average	187,940	75,899	269
SD	219,823	102,494	320
N	272	272	272
261			
Average	173,338	115,855	353
SD	139,482	134,515	328
N	292	292	292
265			
Average	106,859	88,611	137
SD	71,721	67,438	113
N	72	72	72
268			
Average	60,258	31,248	137
SD	43,468	21,133	86
N	146	146	146
273			
Average	211,009	103,219	303
SD	223,672	185,989	317
N	531	531	531
274			
Average	749,996	113,034	419
SD	899,288	115,769	515
N	153	153	153
282			
Average	153,514	34,799	205
SD	540,670	52,127	248
N	229	229	229
286			
Average	60,126	21,766	203
SD	43,444	20,699	219
N	146	146	146
291			
Average	125,900	39,135	266
SD	182,999	68,052	348
N	370	370	370
295			
Average	207,414	68,144	299
SD	277,474	87,996	292
N	190	190	190
313			
Average	246,809	70,225	353
SD	246,577	78,962	328
N	249	249	249
314			
Average	173,134	37,183	363
SD	61,716	28,069	225
N	97	97	97
Total			
Average	235,276	97,243.18	313.58
SD	405,493.4	201,739.1	393.36
N	4414	4414	4414

**Table 7:** Two-sample *t* test with unequal variances, domestically owned versus foreign-owned, by labour productivity

Group	Obs	Mean	SE	SD
Domestically owned	2204	12,685.17	393.61	18,478.91
Foreign-owned	2210	18,410.50	343.6832	16,156.77

Degrees of freedom 4332; *t* = −10.9567

**Table 8:** Two-sample t-test with unequal variances, domestically owned vs foreign-owned, by capital

Group	Obs	Mean	SE	SD
Domestically owned	2204	68,953.98	2723.222	127,846.5
Foreign-owned	2210	125,455.6	5326.524	251,813.6

Degrees of freedom = 4412; *t* = −9.9353

**Table 9:** Two-sample *t* test with unequal variances, domestically owned vs foreign-owned by employees

Group	Obs	Mean	SE	SD
Domestically owned	2204	285.9775	7.381161	346.5217
Foreign-owned	2210	341.1024	9.219415	433.4106

Degrees of freedom = 4412; *t* = −4.6662

## Footnotes

1 Sweden went through a deep crisis in the early 1990s, culminating in a turbulent 1992 when, among other things, Sweden switched from a fixed to a floating exchange rate.
